# Habitat matters – Strong genetic and epigenetic differentiation in *Linum catharticum* from dry and wet grasslands

**DOI:** 10.1002/ece3.6689

**Published:** 2020-08-17

**Authors:** Ellen Pagel, Peter Poschlod, Christoph Reisch

**Affiliations:** ^1^ Ecology and Conservation Biology Institute of Plant Sciences University of Regensburg Regensburg Germany

**Keywords:** calcareous grasslands, ecotypes, epigenetics, habitat differentiation, *Linum catharticum*, litter meadows

## Abstract

Plant species differ in their ecological amplitude, with some species occurring in very different habitats under strongly differentiated environmental conditions. We were interested in to what extent the occurrence of *Linum catharticum* in dry calcareous grasslands (Bromion) and wet litter meadows (Molinion), two habitats on opposing ends concerning, for example, moisture level, is reflected on the genetic and epigenetic level. Using AFLP (amplified fragment length polymorphisms) and MSAP (methylation sensitive amplification polymorphisms) analyses, we studied the genetic and epigenetic variation of *L. catharticum* from calcareous grasslands and litter meadows. From each habitat, we included five study sites with 16 individuals per sampling location. We observed lower genetic than epigenetic diversity, but considerable differentiation among habitats, which was stronger on the genetic than the epigenetic level. Additionally, we observed a strong correlation of genetic and epigenetic distance, irrespective of geographic distance. The dataset included a large portion of fragments exclusively found in individuals from one or the other habitat. Some epigenetic fragments even occurred in different methylation states depending on the habitat. We conclude that environmental effects act on both the genetic and epigenetic level, producing the clear differentiation among plant individuals from calcareous grasslands and litter meadows. These results may also point into the direction of ecotype formation in this species.

## Graphical abstract

In this study, we investigated the genetic and epigenetic variation of *Linum catharticum*. Using AFLP and MSAP analyses, we studied the differences among populations originating from different habitats: dry calcareous grasslands and wet litter meadows. We observed large genetic as well as epigenetic differences among populations from different habitats and conclude that *Linum catharticum* likely forms ecotypes depending on the environmental conditions.

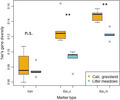

## 1 INTRODUCTION

Through the wide variety of global ecosystems, plant species experience an extraordinary range of environmental conditions among and within habitats (Schulz, Eckstein, & Durka, 2014). Some species, often referred to as habitat specialists, are limited to very specific habitat conditions, like salt marsh or alpine species. This specialization is often considered as a limited niche breadth. Others are known as habitat generalists and have a broader ecological amplitude, enabling them to occur under different habitat conditions (Devictor et al., 2010). The study of habitat specialization and the concept of the ecological niche has been widely under discussion and different concepts have been described (Chase & Leibold, 2003; Devictor et al., 2010). It has been proposed that the occurrence of habitat specialists is governed mainly by environmental processes, while the distribution of generalist species is determined more by dispersal processes (Pandit, Kolasa, & Cottenie, 2009). However, it is likely that intraspecific variation plays a key role in the ability of plant species to grow under specific conditions. Evolutionary mechanisms have led to the adaptation of plant species to varying conditions and phenotypic plasticity enables plant individuals to cope with rapid changes. Many of these processes depend on genetic variation and evolutionary mechanisms, but there is also growing evidence that epigenetic processes play a major role in the response of plant individuals and populations to different or changing environmental conditions, especially on short time scales (Gáspár, Bossdorf, & Durka, 2019; Medrano, Herrera, & Bazaga, 2014).

Different epigenetic mechanisms, such as histone modifications, small RNAs, and DNA methylation, can cause stable alterations in gene expression, while DNA sequences remain unchanged (Bossdorf, Richards, & Pigliucci, 2008; Schulz, Eckstein, & Durka, 2013; Verhoeven, Jansen, van Dijk, & Biere, 2010). The best studied mechanism to date is DNA methylation, which frequently occurs at CG sites in promotor regions in the DNA sequence. The addition of a methyl group to the cytosine molecule often leads to gene silencing, but can also activate gene expression (Bossdorf et al., 2008; Schulz et al., 2013). DNA methylation can be caused via genetic control, environmental influences or by spontaneous epimutations (Richards et al., 2017). Epigenetic modifications can be stably inherited across generations (Gáspár et al., 2019), thus transmitting favorable phenotypic variation to the offspring generations. Epigenetic variation is often correlated with genetic diversity, but several studies also found independent epigenetic variation, which was explained by environmental conditions rather than genetic variation (Bossdorf et al., 2008; Herrera & Bazaga, 2010; Herrera, Medrano, & Bazaga, 2014; Medrano et al., 2014; Richards, Bossdorf, & Verhoeven, 2010; Riddle & Richards, 2002). By the environmental selection of stable epigenetic variants with increased fitness, correlated genetic selection can be guided. However, the magnitude of this potential in wild populations has yet to be studied more intensively (Herrera & Bazaga, 2010).

In recent years, studies of natural populations have been added to the studies of model organisms, like *A. thaliana* or crop species (Heer, Mounger, Boquete, Richards, & Opgenoorth, 2018), broadening our understanding of epigenetic processes under natural conditions. Most recent studies focussed on perennial plant species, for example, clonal *Populus tremuloides* stands (Ahn, Franklin, & Douhovnikoff, 2017), salt marsh perennials (Foust et al., 2016), or a typical grassland species like *Plantago lanceolata* (Gáspár et al., 2019). All these studies found epigenetic differences, which could at least partially be attributed to differences in their local environmental conditions (e.g., flooding frequency and intensity, habitat openness, and herbivory). These results suggest that epigenetic variation is indeed an important mechanism for plants to cope with rapid changes in their environment. However, data from species with really broad ecological amplitudes concerning soil physical or chemical parameters, exceeding the differences mentioned above are, to our best knowledge, still missing.

Epigenetic variation also has great potential and significance in nature conservation and restoration. Local adaptations of plant populations might not be fixed genetically, but (partially) epigenetically, thus adding another concern to exchange of plant material among sites. The origin of plant material for restoration of plant populations or habitats is important, as local adaptations may not be advantageous in new surroundings (Bucharova et al., 2017; McKay, Christian, Harrison, & Rice, 2005). In Germany, seed transfer zones have been established to prevent the mixing of regionally adapted material (Durka et al., 2017). However, species with a broad ecological niche can occur on different habitats and thus be differently adapted within these zones, which may not be visible by the genetic fingerprint alone. Different populations of a generalist species can contain specialized individuals that represent only a part of the broader population's ecological niche (Araújo, Bolnick, & Layman, 2011).

In this study, we were interested in the plant species *Linum catharticum*. This species occurs on a broad range of habitats, reaching from dry to wet grasslands, making it a typical indicator for periodically wet or dry conditions in dry and wet grasslands respectively. Changes in water availability are a key aspect of global change dynamics and have profound effects on phenological and physiological plant processes (Reyer et al., 2013). Therefore, the study of a species growing on both ends of the moisture spectrum in temperate European grasslands will provide some valuable insights into plant response possibilities under future weather extremes. To investigate the genetic and epigenetic variation of *L. catharticum* under natural, but divergent habitat conditions, we included plant individuals from dry calcareous grasslands and wet litter meadows in our data set. Calcareous grasslands are semi‐natural habitats, which are dependent on grazing to maintain their specific species composition (WallisDeVries, Poschlod, & Willems, 2002). They are generally characterized by dry and nutrient poor soils (Dierschke & Briemle, 2002). The vegetation is dominated by, for example, *Bromus erectus* L. (WallisDeVries et al., 2002; Willerding & Poschlod, 2002) and belongs to the union of *Bromion erecti* (Mucina et al., 2016). Litter meadows are dominated by species like *Molinia caerulea* (L.) moench (Poschlod, Baumann, & Karlík, 2009), and these ecosystems are characterized by nutrient poor and wet soils (Dierschke & Briemle, 2002; Poschlod, 2017). The vegetation belongs to the union *Molinion caeruleae* (Mucina et al., 2016). Today calcareous grasslands are typically grazed by sheep and goats, while litter meadows are mown once in the autumn, both as part of conservation management practices. The two grassland types provide habitat for many endangered plant and animal species, like the spring pasqueflower *Pulsatilla vernalis* (Betz, Scheuerer, & Reisch, 2013) or the marsh fritillary *Euphydryas aurinia* (Brunbjerg et al., 2017) and are threatened by habitat fragmentation, intensification and abandonment (Abt, 1991; Poschlod & WallisDeVries, 2002).

We used Amplified Fragment Length Polymorphisms (AFLP) and Methylation Sensitive Amplification Polymorphisms (MSAP) to investigate the genetic and epigenetic structure of individuals of *L. catharticum* from these different habitats. The use of dominant markers is a powerful, quick, and easy tool to study nonmodel species without large reference data bases and gives stable results on the genetic and epigenetic structure of populations (Lele, Ning, Cuiping, Xiao, & Weihua, 2018; Schulz et al., 2013). These methods provide a first step toward understanding the role of genetic and epigenetic variation in *L. catharticum*. By these means, we aimed at investigating one of the basic ecological questions (Bossdorf et al., 2008), whether individuals from the two different habitats showed differences in their genetic and epigenetic variation and how this variation is structured among study sites. We hypothesized that epigenetic variation will show a clear pattern across study sites, while genetic variation within and among sites will be comparably low.

## 2 MATERIALS AND METHODS

### 2.1 Study species

*Linum catharticum* L. occurs as an annual or biennial herb (Ebel & Mühlberg, 1987; Hensen, 2008) and is common within the study region, but locally populations are decreasing due to land use intensification or meadow afforestation (Sebald, Seybold, & Philippi, 1992). Pollination occurs by small dipterous insects (Düll & Kutzelnigg, 2005), but seeds are frequently produced via selfing (Knuth, 1898; Lundgren, Lazaro, & Totland, 2013). The small sticky seeds are dispersed by animals or by hayseed (litter meadows; Poschlod & Biewer, 2005) and also form a long lived seed bank (>100 years) (Fischer, Poschlod, & Beinlich, 1996; Milberg, 1994; Poschlod, Kiefer, Tränkle, Fischer, & Bonn, 1998), but seedling mortality is considered high (Bradshaw & Doody, 1978). *L. catharticum* mainly occurs on calcareous substrate with a wide range of moisture levels (Oberdorfer, Schwabe, & Müller, 2001; Sebald et al., 1992).

### 2.2 Study sites and sampling

To investigate the genetic and epigenetic differences due to habitat, we collected plant samples from two habitats on opposing ends of the moisture spectrum: calcareous grasslands and litter meadows. In a previous study (Lehmair, Pagel, Poschlod, & Reisch, unpublished), we analyzed the genetic variation of *L. catharticum* from 19 calcareous grasslands across the Swabian Alb and found intermediate levels of genetic diversity and low levels of genetic differentiation among study sites. We did not detect any indication for isolation by distance, despite the considerable maximum distance of 85 km among sites. Some of these calcareous grasslands were therefore included in the present study and the genetic and epigenetic diversity within these sites, compared with individuals growing on litter meadows in the Allgäu region.

The differences among calcareous grasslands and litter meadows are visible on the biotic and abiotic level. To show these differences data on the vegetation structure and local soil conditions were collected from five 2 × 2 m plots at each study site. Next to the abundance of different species Ellenberg indicator values (EIV) (Ellenberg & Leuschner, 2010) were calculated for each study side. The EIV were used to describe the abiotic conditions at the site in combination with basic soil analysis.

Study sites were selected, based on the availability of sufficient numbers of plant individuals and five sites were chosen for each habitat, with distances of 7 to 30 km between study sites within the same habitat. Five study sites of *L. catharticum* were located on calcareous grasslands, which were compared with five study sites of the more wet litter meadows (Table 1, Figure S1). Distances between sampling sites of different habitats ranged between 56 and 95 km. At each study site 16 plant samples were collected (min. distance between samples: 5 m, average distance: 25 m) in individual plastic bags and frozen in liquid nitrogen. Sampling took place during the early phase of flowering, so samples were from comparable life stages. The samples were stored in the lab at −20°C until DNA extraction.

**Table 1:** Number and names of the analyzed populations, their respective habitat, the geographic location they are situated in, as well as the number of analyzed individuals per population

Nr	Name	Habitat	Lat	Lon	*N*
C1	Bichishausen	Calcareous grassland	48.3349	9.5013	16
C2	Justingen	Calcareous grassland	48.4034	9.6905	16
C3	Büchelesberg	Calcareous grassland	48.3080	9.7247	16
C4	Hohenstein	Calcareous grassland	48.3202	9.3159	16
C5	Gomadingen	Calcareous grassland	48.3911	9.3770	16
L1	Arrisried	Litter meadow	47.7536	9.8787	10
L2	Weitershofen	Litter meadow	47.8169	9.8996	13
L3	Argen	Litter meadow	47.6702	10.0746	16
L4	Rotheidlen	Litter meadow	47.7320	9.7161	16
L5	Nitzenweiler	Litter meadow	47.6077	9.6367	15

### 2.3 Molecular analyses

From the frozen plant material, DNA was extracted using the CTAB protocol by Rogers and Bendich (1994), modified by Reisch (2007). DNA extracts were diluted with water to a standardized concentration (7.8 ng/µl) and used for further analysis. In a first step samples were analyzed using Amplified Fragment Length Polymorphisms (AFLP) according to the protocol by Reisch (2008) adapted from Vos et al. (1995). The same DNA samples were then used in a Methylation Sensitive Amplified Polymorphism analysis (MSAP), as described by Salmon, Clotault, Jenczewski, Chable, and Manzanares‐Dauleux (2008) and Schulz et al. (2013). This procedure is based on the same protocol as the AFLP analysis, but uses each sample in two separate reactions, starting with the DNA restriction step. Instead of *MseI* which is used for AFLP analysis, two other restriction enzymes, which are so called isoschizomers (*HpaII* and *MspI*), are paired with *EcoRI*. Due to their specific methylation sensitivity they allow for differentiation between different methylation states within their restriction site. (For further methodological details see (Schulz et al., 2013).

Prior to both AFLP and MSAP analysis suitable primer combinations were screened and for each marker type three combinations chosen for this analysis (Table S1).

Individuals that resulted in unclear band patterns were repeated once and then omitted from the final data set, thus resulting in 80 samples for the calcareous grasslands and 70 samples from litter meadows.

For both the AFLP and MSAP analyses, a genotyping error rate following the procedure of Bonin et al. (2004) was estimated by repeating the analysis of 10% of the studied individuals (16 individuals) and comparing resulting band patterns. This procedure resulted in a genotyping error rate of 2.52% in the AFLP analysis and of 1.38% in the MSAP analysis. We therefore conclude that the observed differences among individuals and study sites are due to actually present molecular differences and not due to methodical errors.

### 2.4 Data analysis

AFLP and MSAP fragment data were analyzed separately, using the software Bionumerics 7.6.3 (Applied Maths) to create a binary matrix for each dataset, representing the presence and absence of a given fragment for each individual. The AFLP dataset included 158 fragments, while the MSAP dataset comprised 337 fragments. The 0/1 matrix of the MSAP data set was then scored for unmethylated (Epi_u) and methylated (Epi_m) and hemimethylated (Epi_h) epiloci using the scoring method “Mixed‐Scoring 2” as proposed by Schulz et al. (2013), using the R‐script “MSAP_calc” (Durka, 2012). This scoring procedure then resulted in a binary matrix for each epilocus type (302 Epi_u loci, 282 Epi_m loci and 222 Epi_h loci). However, hemimethylated loci were only present in very few individuals and were omitted from further analyses. Using the implemented PCoA Analysis in the “MSAP_calc” script all remaining datasets were analyzed thus.

For the three binary matrixes (AFLP, Epi_u, Epi_m) genetic variation within study sites, expressed by Nei's Gene Diversity (H = 1– Σ (p_i_) ^2^) was calculated using PopGene 32 (Yeh, Yang, Boyles, Ye, & Mao, 1997). To test for differences among habitats in the height of their genetic diversity we used a Wilcoxon–Mann–Whitney *U*‐test.

To explore genetic and epigenetic differentiation, a hierarchical Analysis of Molecular Variance (AMOVA) (Excoffier, Smouse, & Quattro, 1992), based on pairwise Euclidean distances between samples, was calculated applying GenAlEx 6.5 (Peakall & Smouse, 2012). The two habitats were used as regions in this analysis.

To study the population structure a Bayesian cluster analysis was performed with Structure 2.3.4 (Pritchard, Wen, & Falush, 2010) separately for all four marker types. To calculate the most likely number of groups we used a burn‐in of 10,000 iterations and 100,000 MCMC simulations with K set between 1 and 12. For each K analysis was run 20 times. We set POPFLAG to 0 and used an admixture model, assuming allele frequencies to be correlated among populations. The web tool “Structure Harvester” (Earl & vonHoldt, 2012) was used to summarize the results. Following the method of Evanno, Regnaut, and Goudet (2005) we used the highest ΔK value to determine the best estimate of K.

The use of simple and partial Mantel tests has been discussed in the literature as being only partially appropriate for studying the correlation of genetic, geographic and environmental distances (Bradburd, Ralph, & Coop, 2013; Guillot & Rousset, 2013; Legendre, Fortin, & Borcard, 2015). Therefore, we based our correlation analyses on the example of Lele et al., (2018) and used simple and partial Mantel tests as well as multiple matrix regressions with randomization (MMRR) analyses. Based on the genetic and epigenetic distance values (ɸ_PT_ values), produced by the AMOVA, and the Euclidean geographic distance among sampling sites we performed simple Mantel tests using GenAlEx and partial Mantel tests using the “vegan” package (Oksanen et al., 2019), to test for the correlations of genetic and epigenetic distance with geographic distance and the respective other (epi)genetic distances. Additionally, analogous to Lele et al., (2018) we used the MMRR function provided by Wang, (2013) using 9,999 permutations, to test for correlations of genetic and geographic distances, while controlling for epigenetic distance.

All analyses were conducted in R Studio 1.1.423 (RStudio Team, 2016), if not otherwise specified.

## 3 RESULTS

### 3.1 Environmental differences among calcareous grasslands and litter meadows

The vegetation structure of the two studied habitats differed in their grass, legume and herb cover. While calcareous grasslands were more dominated by herbaceous species, grasses dominated the vegetation cover in litter meadows (Table S2). The two habitats showed large differences in water and nutrient availability. While calcareous grasslands showed dry conditions, litter meadows were in wet conditions. Additionally, litter meadows showed more acidic soils than calcareous grasslands. The same pattern was visible for the nutrient availability, calcareous grasslands were less limited in their available nutrients than litter meadows (Table S3); however, both habitats would still be considered nutrient poor compared to other grassland types.

### 3.2 Genetic and epigenetic diversity and structure

**Figure 1:**
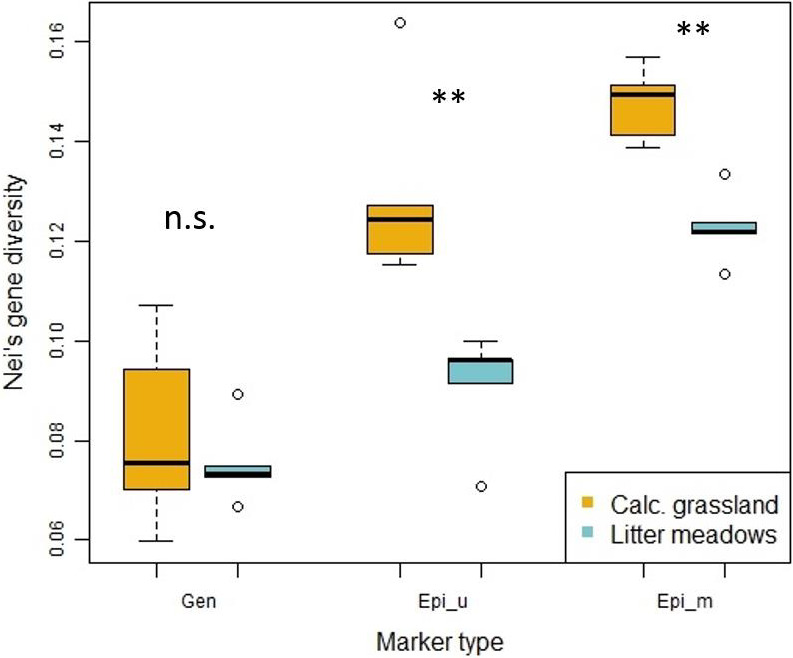
Nei's gene diversity separated by marker type, in calcareous grasslands (orange) and in litter meadows (blue). The diversity for Epi_u (epigenetic variation of unmethylated epiloci) and Epi_m (epigenetic variation of methylated epiloci) is significantly different among habitats (Epi_u: *p* =< 0.01; Epi_m: *p* =< 0.01)

Genetic diversity was generally low, with an average over all study sites of 0.078 (±0.013) and did not differ significantly among habitats. However, genetic diversity within calcareous grassland study sites showed a higher heterogeneity, while for litter meadows the study sites showed very similar genetic diversity. Epigenetic diversity for unmethylated and methylated epiloci differed significantly among habitats, with individuals from litter meadows showing lower genetic and epigenetic diversity, than those from calcareous grasslands. (Figure 1, Table S4).

The datasets showed many fragments to be specific for one of the two habitats. The AFLP dataset included 33 fragments private to calcareous grasslands, while 25 fragments were only found in individuals from litter meadows. The MSAP dataset also showed large numbers of epiloci exclusively found in individuals from one or the other habitat (CG: Epi_u 62, Epi_m 85, LM: Epi_u 44, Epi_m 31). Additionally, we checked for epiloci, which were found as methylated in one habitat, while it occurred unmethylated in the other and vice versa. In total, we found 13 of these fragments, of which seven were primarily methylated (on average 93%) in litter meadow individuals and mainly unmethylated (on average 92%) in calcareous grassland individuals. The other six fragments showed methylation in calcareous grasslands (on average 95%), while they appeared primarily unmethylated in litter meadows (on average 97%).

Differentiation among habitats was generally very high. Genetic differentiation among habitats was 80%. Epigenetic differentiation was lower among habitats than genetic differentiation (Epi_u: 62%, Epi_m: 57%) (Table 2).

**Table 2:** Results of the Three‐Level AMOVAs given as the genetic variation among habitats, among and within the respective populations for each marker type

	*df*	% variation	ɸ‐statistics
Genetic variation for Gen			
Among Habitats	1	80	0.835***
Among Pops	8	3	
Within Pops	140	17	
Epigenetic variation for Epi‐u			
Among Habitats	1	62	0.663***
Among Pops	8	5	
Within Pops	140	34	
Epigenetic variation for Epi‐m			
Among Habitats	1	57	0.573***
Among Pops	8	3	
Within Pops	140	39	

Note

Epi‐m, epigenetic variation of methylated epiloci; Epi‐u, epigenetic variation of unmethylated epiloci.

*** *p* = .001.

**Figure 2:**
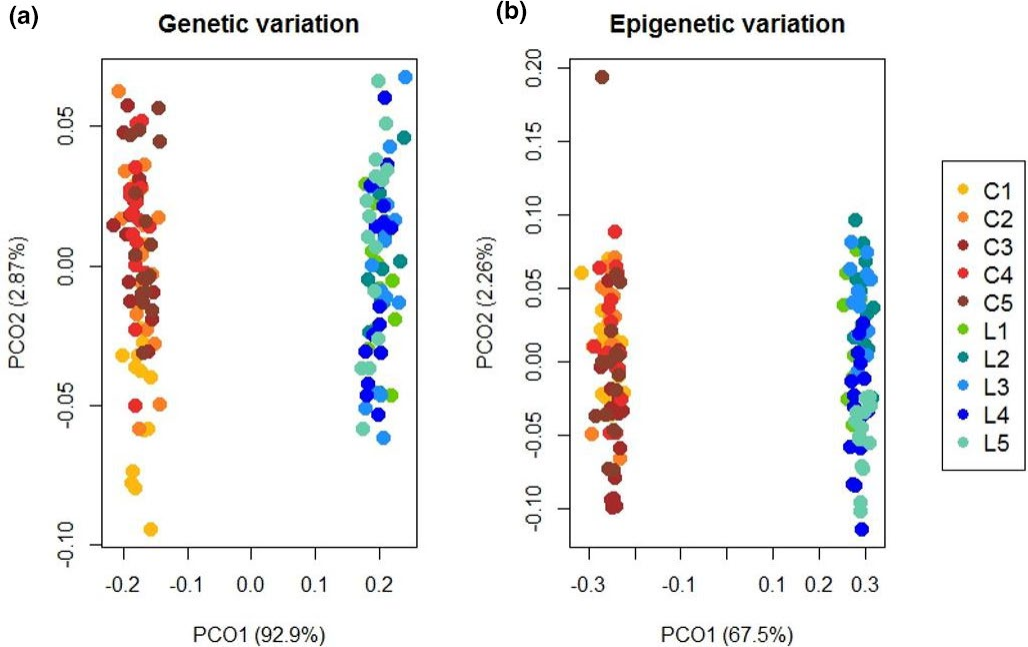
Principal Coordinate Analysis of the genetic dataset (a) and epigenetic dataset (b). The two habitats (C – calcareous grasslands, L – litter meadows) are clearly separated in two groups, on the genetic and epigenetic level

These results are further illustrated by the Principal Coordinate Analysis, which showed the two habitats as clearly separated, both on the genetic (Figure 2a) and epigenetic level (Figure 2b). The first axis explained 92.9% of the variation within the genetic dataset, while the first axis for the epigenetic dataset explained 67.5%. The second axis explained around two percent of the variation in both datasets, showing the high within‐habitat similarity of the studied sites. The Bayesian cluster analysis gave *K* = 2 as the most likely number of groups for all marker types (DeltaK values: Gen: 706.04, Epi_u: 408.71, Epi_m: 346.35), which represented the two habitats (Figure S2), additionally supporting the above described results.

**Figure 3:**
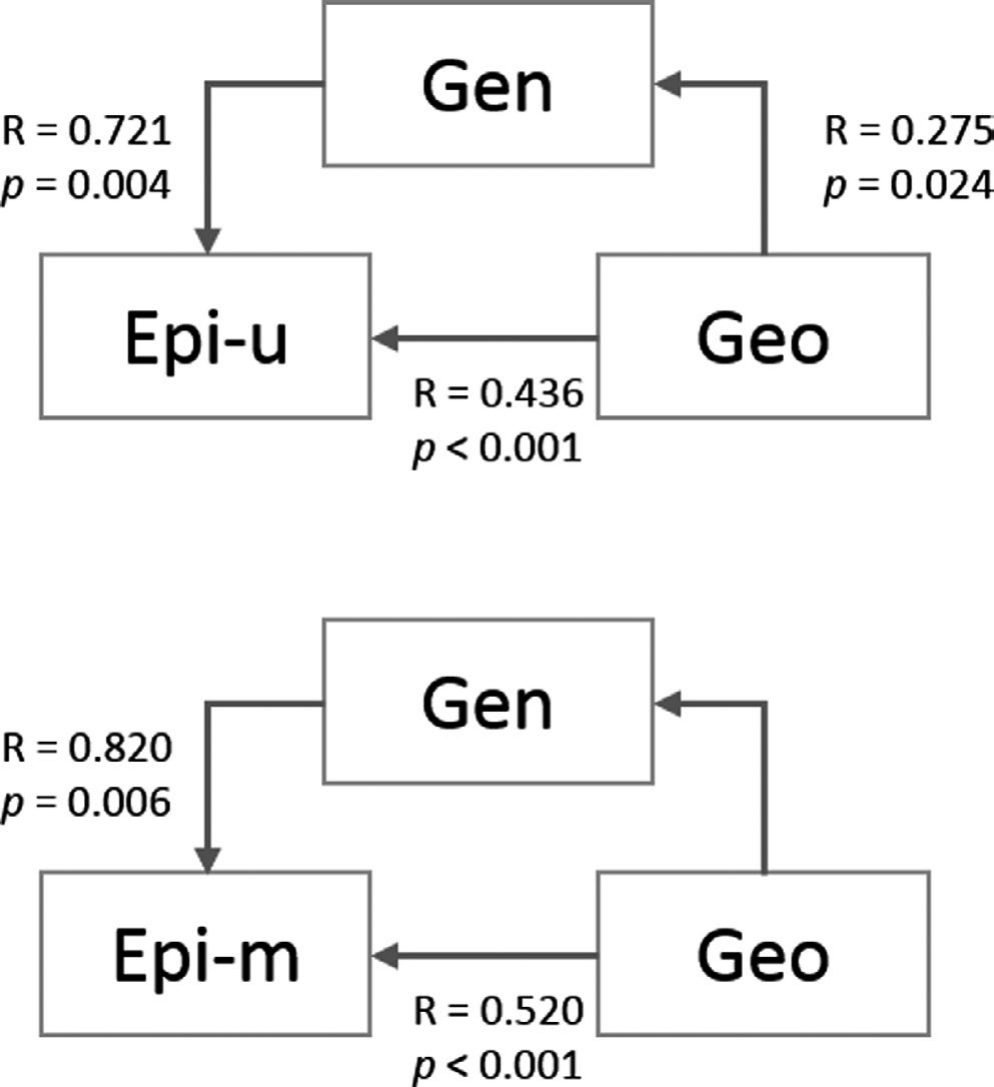
Genetic and epigenetic correlations to variation in geographic distance using partial Mantel tests with the Euclidean genetic and epigenetic distance matrices and geographical distance matrices across all populations. The correlations between genetic and epigenetic distance were calculated in separate partial Mantel tests. (Gen = genetic variation, Epi‐u = epigenetic variation of unmethylated epiloci, Epi‐m = epigenetic variation of methylated epiloci, Geo = geographical distance, NS = not significant, r = correlation coefficient and respective *p*‐value)

Simple Mantel tests showed strong and significant correlations between genetic and epigenetic distances, as well as of all marker types with geographic distance (Table 3). Partial Mantel test and the MMRR analysis showed, that the correlations between genetic and epigenetic distances were much more pronounced than the impact of geographic distance for unmethylated and methylated epiloci. For the correlation of genetic distance with epigenetic distance of methylated loci, the correlation with geographic distance was not significant, even though they were strongly correlated in the simple Mantel test (Figure 3 and Table 4).

**Table 3:** Results of the Mantel tests conducted for genetic (Gen) and epigenetic (Epi‐u = epigenetic variation of unmethylated epiloci, Epi‐m = epigenetic variation of methylated epiloci) distance with geographic distance (Geo) and the respective other (epi)genetic distances. Above diagonal is the *R* ^2^ value and the respective *p*‐value below the diagonal

	Gen	Epi‐u	Epi‐m	Geo
Gen	x	0.944	0.964	0.892
Epi‐u	0.006	x	0.986	0.905
Epi‐m	0.001	0.001	x	0.921
Geo	0.003	0.001	0.002	x

**Table 4:** Summary of the multiple matrix regression analysis with randomization (MMRR) relating the genetic distance matrix with geographic and epigenetic distance matrices

Differentiation Matrix	Epigenetic Matrix used	Overall Regression		Linear predictor matrices			
				Geographical Distance		Epigenetic Distance	
		*F*	*p*	Coefficient	*p*	Coefficient	*p*
Gen	Epi‐u	381.237	.0011	0.0024	.024	0.967	.0018
Gen	Epi‐m	570.13	<.001	0.0003	.75	1.247	.0014

Note

Epi‐m, epigenetic variation of methylated epiloci; Epi‐u, epigenetic variation of unmethylated epiloci.

## 4 DISCUSSION

It has been frequently reported in recent years, that epigenetic mechanisms may contribute significantly to the adaptive potential of plant individuals in natural populations (Bossdorf et al., 2008; Gáspár et al., 2019; Richards et al., 2010) and several studies have found evidence for habitat or environment specific epigenetic differences among populations (Ahn et al., 2017; Lira‐Medeiros et al., 2010; Richards, Schrey, & Pigliucci, 2012).

In our study, we found comparably low levels of genetic diversity within study sites. With a mean Nei's gene diversity of 0.078 over all sites, genetic diversity was lower than we would have expected, even for a mainly self‐pollinated species (Reisch & Bernhardt‐Römermann, 2014). In the previous study of *L. catharticum* from 19 calcareous grasslands, mean genetic diversity was estimated at 0.155 (Lehmair et al., unpublished), which is more in accordance with the values expected for rather common species. However, the present dataset includes many private fragments, only present in study sites from one or the other habitat type. The abundance of these fragments decreased the estimated genetic diversity within the different sampling sites. The epigenetic datasets also included many private fragments. However, epigenetic diversity for unmethylated and methylated epiloci was still higher than genetic diversity. This finding is consistent with the results of other studies (Lira‐Medeiros et al., 2010; Richards et al., 2012; M.‐Z. Wang, Li, Li, & Yu, 2019), which frequently also found higher epigenetic than genetic diversity in their studied nonmodel species. The low genetic diversity in these studies can be explained also by their study design of choosing clonal species. As a mainly selfing species (Knuth, 1898; Lundgren et al., 2013) *L. catharticum* recombination events can be considered as rare, thus explaining the low genetic diversity. Epigenetic diversity is influenced by environmental conditions and spontaneous epimutations can also contribute to the observed higher epigenetic than genetic diversity (Wang et al., 2019). Genetic and epigenetic differentiation was high among individuals from the two different habitats. Even though genetic diversity was lower than epigenetic diversity, differentiation was higher on the genetic than on the epigenetic level. In our previous study, on *L. catharticum* from calcareous grasslands across the Swabian Alb (max distance among sites: 85 km) variation among study sites was estimated at 8% (Lehmair et al., unpublished). These findings present tremendous differences compared to the levels of differentiation found between the two different habitats. This indicates, that geographic distance alone cannot explain the strong genetic and epigenetic differentiation found here between habitats, with maximum distances of 95 km. The maximum geographic distance between sites is similar to our previous study; however, differentiation was much higher in the present study. Both the MMRR analysis and the partial Mantel tests showed that the correlation of genetic and epigenetic variation was quite marked and that the influence of geographic distance was of minor importance. These results are comparable to Lele et al., (2018), who also found a strong correlation of the genetic and epigenetic variation and low correlations with geographic distance in *Vitex negundo var. heterophylla* (Chinese chastetree), even though the maximum distance between study sites was 150 km, spanning an even wider geographical range than our study. Lele et al., (2018) also found correlations between epigenetic variation and phenotypic diversity in their study; however, local adaptation to divergent habitat conditions was mainly attributed to genetic variation.

In our dataset, genetic and epigenetic fingerprints were strongly associated with the habitat of origin. Additionally, we found a number of MSAP fragments present in both habitats, but which had different methylation states in each of them (methylated in calcareous grasslands and unmethylated in litter meadows). Environmental disturbances are expected to be higher in calcareous grasslands, with multiple stress factors, for example, water limitation, grazing and trampling, nutrient limitations, contributing to a generally more heterogeneous habitat (WallisDeVries et al., 2002), also expressed by the soil conditions of the studied grasslands. In litter meadows, there is typically only one major disturbance event, that is, mowing during autumn (Kapfer, 1995). These environmental differences can therefore, explain the divergence on the genetic and epigenetic level found here and also the higher epigenetic variation found in individuals from calcareous grasslands.

An influence of environmental conditions on epigenetic variation was found in several studies (Foust et al., 2016; Medrano et al., 2014; Richards et al., 2012; Verhoeven et al., 2010). Verhoeven et al., (2010) found an increase in epigenetic variation within stress treatments in *Taraxacum officinale*, especially in an herbivory and pathogen defense trigger treatment. Under high simulated herbivory or pathogen pressure epigenetic variation increased. A connection between grazing intensity and epigenetic variation was also found for *Plantago lanzeolata* (Gáspár et al., 2019). Herbivory by large animals is an important stressor associated with calcareous grasslands, which is not present in litter meadows in an equal way. Additionally, *L. catharticum* populations have been found to decrease in fitness parameters under high trampling intensities (Bradshaw & Doody, 1978). This suggests that the observed differences in genetic and epigenetic diversity, differentiation and also the marker specific differences can be attributed not only to geographic distance, but maybe more importantly to the difference in disturbance intensity between the habitats. Next to disturbance, local soil conditions with regards to moisture and pH were significantly different between the two habitats and might also contribute to the observed genetic and epigenetic differences. Changes in water levels have been found to result in quick alterations in the methylation patterns of *Alternanthera philoxeroides* (Gao, Geng, Li, Chen, & Yang, 2010), suggesting that the genetic and epigenetic differences observed in *L. catharticum* might also be driven by the differences in soil moisture.

Some studies have found environmental processes to influence genetic and epigenetic diversity independently (Verhoeven et al., 2010), but many studies also found a strong correlation of genetic and epigenetic diversity (Dubin et al., 2015; Herrera & Bazaga, 2010; Kawakatsu et al., 2016; Robertson, Schrey, Shayter, Moss, & Richards, 2017; Schulz et al., 2014; Shan et al., 2012; M.‐Z. Wang et al., 2019). The genetic and epigenetic variation in this study was strongly correlated, suggesting parallel processes, both on the genetic, and thus evolutionary, level and on the epigenetic level, indicating different epigenotypes or ecotypes in the different habitats. The extent to which these differences are visible on differently expressed genes and phenotypes would need to be assessed via further more detailed studies, for example, with common garden and crossing experiments. More advanced molecular tools might help to determine which genes are differently regulated within the two habitats and which environmental factors are truly the driving force behind the observed patterns. The strong genetic differentiation could be cautiously interpreted as an indication of different ecotype being present in the different ecosystems. Different subspecies have previously proposed for *L. catharticum*, based on differences in morphology, but scientific evidence for the parallel occurrence of the two described subspecies in the study region is not available (Sebald et al., 1992). As we did not collect data on the morphology of the studied individuals we cannot support or oppose the possible occurrence of different subspecies.

As Herrera and Bazaga, (2010) already discussed, to understand the potential of epigenetic variation in adaptive and evolutionary processes, we need more studies in natural contexts. We therefore conclude that the results presented here give some indications on the magnitude of differences possible within a species and a comparably restricted geographical setting and provides a starting point for future research, especially with regards to future environmental changes and the potential of epigenetic processes to facilitate plant population fitness under changing conditions.

## CONFLICT OF INTEREST

The authors declare that they have no conflict of interest.

## AUTHOR CONTRIBUTION

**Ellen Pagel:** Formal analysis (lead); Investigation (lead); Project administration (equal); Writing‐original draft (lead). **Peter Poschlod:** Funding acquisition (lead); Supervision (equal); Writing‐review & editing (equal). **Christoph Reisch:** Project administration (equal); Resources (equal); Supervision (equal); Writing‐review & editing (equal).

## Supporting information

Supplementary Material

## Data Availability

AFLP and MSAP fingerprint data can be downloaded from Dryad https://doi.org/10.5061/dryad.8gtht76mj
