# Supplementary material for: Habitat matters – Strong genetic and epigenetic differentiation in Linum catharticum from dry and wet grasslands
Source: Ecol Evol. 2020 Aug 17;10(18):10271–80. doi: 10.1002/ece3.6689 (PMC7520193; doi:10.1002/ece3.6689)
Supplement: Supplementary file 1 — Supplementary Material [file ECE3-10-10271-s001.docx]

**Supplemental Information for:**

**Habitat matters – strong genetic and epigenetic differentiation**

**in *L. catharticum* from dry and wet grasslands**

Ellen Pagel, Peter Poschlod, Christoph Reisch

University of Regensburg, Institute of Plant Sciences, Ecology and Conservation Biology

**Figure S1** Map of all study sites. Calcareous grasslands (C1-C5) were located on the Swabian Alb and litter meadows (L1-L5) in the Allgäu region. Distances among study sites between the two habitats ranged between 56 and 95 km, while distances within the habitats ranged between 7 and 33 km.


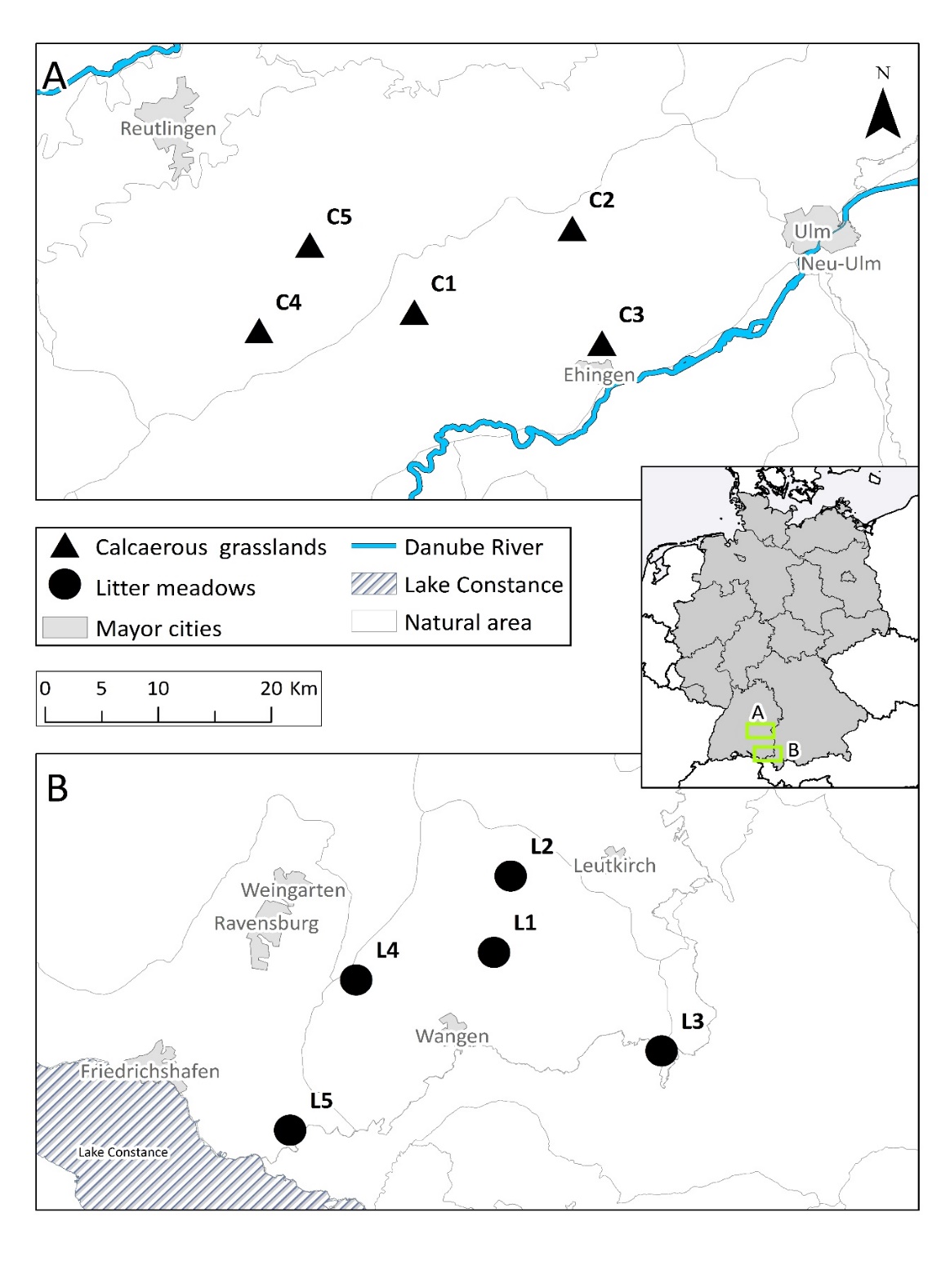


**Figure S2** Results of the Bayesian Cluster Analysis for all marker types. (Gen – genetic variation, Epi_u – unmethylated epiloci, Epi_m – methylated epiloci). DeltaK values: Gen: 706.04, Epi_u: 408.71, Epi_m: 346.35.

**
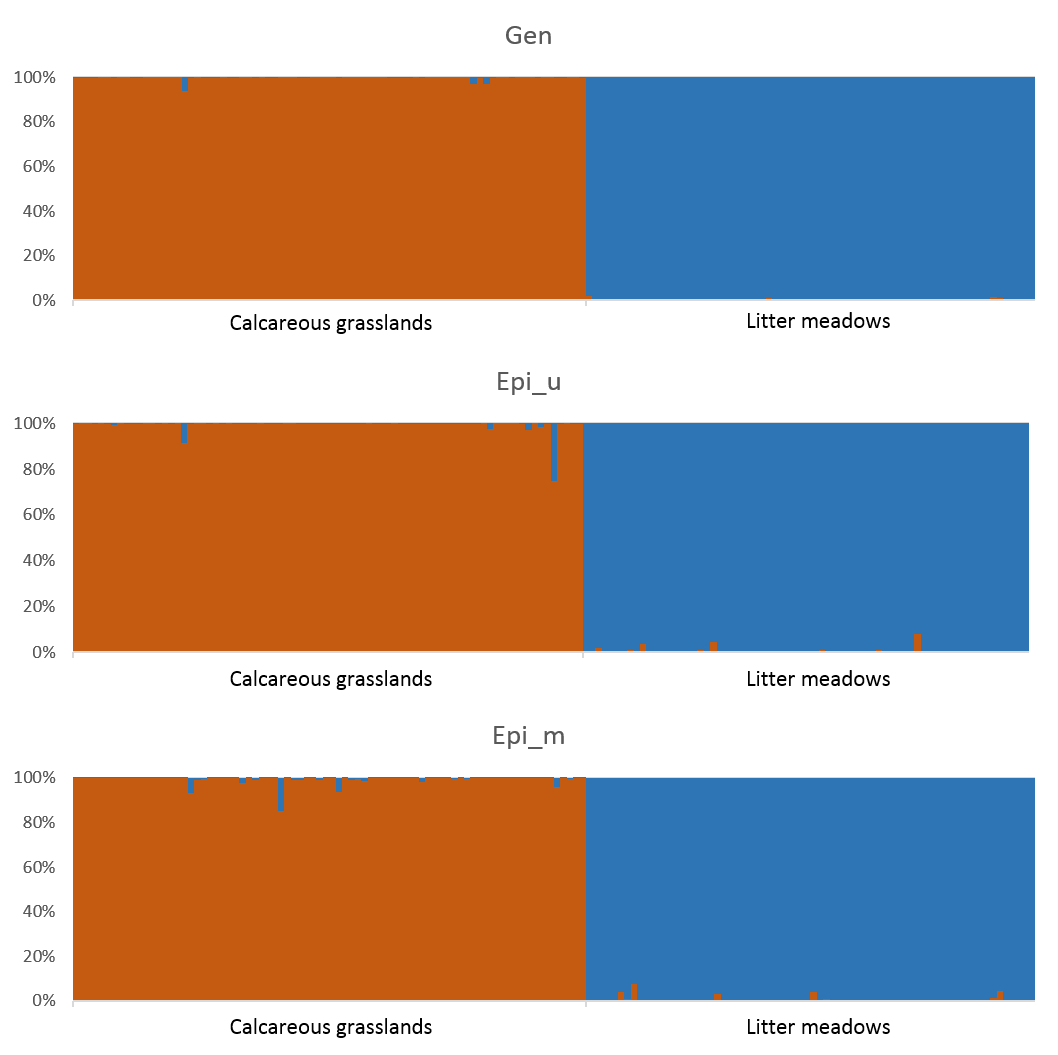
**

**Table S1** The three primer combinations for each Marker type used for each species with the respective fluorescent dye.

| AFLP | Primer combination |
| --- | --- |
| *D2* | M-CTC – E-AAC |
| *D3* | M-CTA – E-AGG |
| *D4* | M-CAA – E-ACA |
|  |  |
| MSAP |  |
| *D2* | H/M-TCCA – E-AAC |
| *D3* | H/M-AAT – E-AAG |
| *D4* | H/M-TCAA – E-ACT |

**Table S2** Vegetation Structure represented by the different percentage covers of vascular plants (VP), mosses (Moss), plant litter (Lit), open soil (O-Soil), grasses (Grass), legumes (Leg) and herbaceous species (Herb) for all study sites. Estimates were calculated from five plots (2x2 m) per study site. The given p-value is based on t-tests.

| **ID** | **VP** | **Moss** | **Lit** | **O-Soil** | **Grass** | **Leg** | **Herb** |
| --- | --- | --- | --- | --- | --- | --- | --- |
| C1 | 79.0 | 43.0 | 3.2 | 7.6 | 28.0 | 2.0 | 51.0 |
| C2 | 82.0 | 81.0 | 3.6 | 0.4 | 34.0 | 5.8 | 42.2 |
| C3 | 79.0 | 57.0 | 10.0 | 7.4 | 32.8 | 12.0 | 34.2 |
| C4 | 83.0 | 78.0 | 5.0 | 1.0 | 39.0 | 3.4 | 40.6 |
| C5 | 88.0 | 88.0 | 9.4 | 0.5 | 31.0 | 6.6 | 50.4 |
|  |  |  |  |  |  |  |  |
| L1 | 73.0 | 69.0 | 9.6 | 1.0 | 55.0 | 0.0 | 17.0 |
| L2 | 79.0 | 43.0 | 22.0 | 1.6 | 63.0 | 0.0 | 16.0 |
| L3 | 76.0 | 59.0 | 10.0 | 2.2 | 40.0 | 3.0 | 34.0 |
| L4 | 79.5 | 62.0 | 11.1 | 2.6 | 52.5 | 1.2 | 26.5 |
| L5 | 80.0 | 71.0 | 1.8 | 1.6 | 41.0 | 1.2 | 38.0 |
|  |  |  |  |  |  |  |  |
| p-value | n.s. | n.s. | n.s. | n.s. | 0.006 | 0.027 | 0.013 |

**Table S3** Soil characteristics of the studied grasslands expressed as the water holding capacity (WHC) [%], the pH measured in CaCl2, phosphorous content [g/kg], potassium content [g/kg] and Carbon/Nitrogen ratio (C/N). Additionally the Ellenberg Indicator values for soil moisture (F), nutrient availability (N) and soil reaction (R) is given. The given p-value is based on t-tests.

| **ID** | **WHC** | **pH** | **P** | **K** | **C/N** | **F** | **N** | **R** |
| --- | --- | --- | --- | --- | --- | --- | --- | --- |
| **C1** | 53.08 | 7.10 | 10.92 | 115.28 | 19.84 | 4.10 | 4.26 | 7.73 |
| **C2** | 92.26 | 5.78 | 16.78 | 111.04 | 13.92 | 4.50 | 4.49 | 7.65 |
| **C3** | 63.32 | 7.08 | 31.36 | 195.16 | 28.88 | 4.28 | 4.63 | 7.75 |
| **C4** | 104.50 | 6.96 | 31.00 | 176.88 | 20.58 | 4.42 | 4.72 | 7.75 |
| **C5** | 85.70 | 6.76 | 12.20 | 53.84 | 17.46 | 4.40 | 4.41 | 7.59 |
|  |  |  |  |  |  |  |  |  |
| **L1** | 80.15 | 5.21 | 43.38 | 70.86 | 17.64 | 7.61 | 2.87 | 6.18 |
| **L2** | 71.13 | 4.09 | 20.90 | 54.27 | 15.74 | 6.94 | 2.45 | 4.87 |
| **L3** | 87.98 | 5.32 | 24.94 | 44.66 | 12.17 | 7.40 | 3.01 | 5.52 |
| **L4** | 82.94 | 5.51 | 33.14 | 84.27 | 14.97 | 7.25 | 2.61 | 6.82 |
| **L5** | 77.60 | 5.09 | 28.59 | 21.50 | 14.43 | 7.68 | 2.84 | 6.78 |
|  |  |  |  |  |  |  |  |  |
| **p-value** | n.s. | 0.001 | n.s. | 0.026 | n.s. | < 0.001 | < 0.001 | 0.002 |

**Table S4** Genetic diversity of all studied populations given as Nei’s gene diversity for all obtained marker types (Gen, Epi-u, Epi-m,) and the respective mean per habitat and over all populations. The p-value for the Wilcoxon-Mann-Whitney-U-test is given (* significant).

| Nr. | Habitat | *Gen* | *Epi-u* | *Epi-m* |
| --- | --- | --- | --- | --- |
| C1 | CG | 0.094 | 0.115 | 0.139 |
| C2 | CG | 0.107 | 0.127 | 0.157 |
| C3 | CG | 0.060 | 0.124 | 0.151 |
| C4 | CG | 0.070 | 0.118 | 0.141 |
| C5 | CG | 0.076 | 0.164 | 0.150 |
|  | **Mean all CG** | **0.081** | **0.130** | **0.148** |
|  | SD | 0.017 | 0.018 | 0.007 |
| L1 | LM | 0.073 | 0.091 | 0.091 |
| L2 | LM | 0.089 | 0.096 | 0.096 |
| L3 | LM | 0.075 | 0.071 | 0.071 |
| L4 | LM | 0.067 | 0.100 | 0.100 |
| L5 | LM | 0.073 | 0.096 | 0.096 |
|  | **Mean all LM** | **0.075** | **0.091** | **0.123** |
|  | SD | 0.007 | 0.010 | 0.006 |
| Mean over all pop. | | **0.078** | **0.110** | **0.135** |
| Standard deviation | | 0.013 | 0.024 | 0.014 |
| p-value | | 0.545 | 0.0078* | 0.0078* |
